# Instrumented Pre-Hospital Care Simulation Mannequin for Use in Spinal Motion Restrictions Scenarios: Validation of Cervical and Lumbar Motion Assessment

**DOI:** 10.3390/s24041055

**Published:** 2024-02-06

**Authors:** Camille Martin, Patrick Boissy, Mathieu Hamel, Karina Lebel

**Affiliations:** 1 Faculty of Engineering, Department of Electrical Engineering, Université de Sherbrooke, Sherbrooke, QC J1K 2R1, Canada; 2 Research Center on Aging, CIUSSS Estrie CHUS, Sherbrooke, QC J1H 4C4, Canada; patrick.boissy@usherbrooke.ca (P.B.); mathieu.hamel2@usherbrooke.ca (M.H.)

**Keywords:** mid-fidelity mannequin, motion assessment validation, spinal motion assessment, spinal motion restriction, spine model

## Abstract

Background: A mid-fidelity simulation mannequin, equipped with an instrumented cervical and lumbar spine, was developed to investigate best practices and train healthcare professionals in applying spinal motion restrictions (SMRs) during the early mobilization and transfer of accident victims with suspected spine injury. The study objectives are to (1) examine accuracy of the cervical and lumbar motions measured with the mannequin; and (2) confirm that the speed of motion has no bearing on this accuracy. Methods: Accuracy was evaluated by concurrently comparing the orientation data obtained with the mannequin with that from an optoelectronic system. The mannequin’s head and pelvis were moved in all anatomical planes of motion at different speeds. Results: Accuracy, assessed by root-mean-square error, varied between 0.7° and 1.5° in all anatomical planes of motion. Bland–Altman analysis revealed a bias ranging from −0.7° to 0.6°, with the absolute limit of agreement remaining below 3.5°. The minimal detectable change varied between 1.3° and 2.6°. Motion speed demonstrated no impact on accuracy. Conclusions: The results of this validation study confirm the mannequin’s potential to provide accurate measurements of cervical and lumbar motion during simulation scenarios for training and research on the application of SMR.

## 1. Introduction

Spinal cord injuries (SCIs) are caused by traumatic events such as traffic accidents, sports, and falls, and result in motor, sensory, or autonomic dysfunctions [1,2]. Zileli et al. reports that 46% of SCIs occur at the cervical level, whereas 24% occur at the lumbar level [3]. When SCI is suspected, health professionals must apply spinal motion restriction (SMR) to transfer and transport the patient to a medical centre. SMR techniques aim at reducing undesired movement of the spine by keeping the head of the patient aligned with their trunk and their trunk aligned with their pelvis. SMR technique training is essential to minimize secondary damage to the spine.

SMR training is usually performed with simulated patients or low-fidelity mannequins. In either case, the biomechanics and the compliance of an unconscious person are not well reproduced. It may thus be harder to transfer learnings from training to real-life situations. Furthermore, there are no objective measures of the quality and effectiveness of the SMR. Indeed, manoeuvres and techniques used are mostly assessed subjectively by expert-trainer observations or from the simulated patient. This is a concern, especially as it was demonstrated that the validity of subjective performance scores from both rescuers and a simulated patient in a context of SMR is poor compared with objective measures of motion [4]. Providing immediate or deferred objective feedback on the quality and effectiveness of SMR applied during pre-hospital care scenarios by healthcare professionals could help to identify the most effective technique in addition to improving SMR techniques’ execution [5,6,7].

Motion capture technologies, such as optoelectronic systems, inertial systems, and electromagnetic systems, can be used to assess motion objectively [8]. Optoelectronic systems are commonly used in sports and motion sciences as well as in the film-making industry. Briefly, markers are placed on anatomical landmarks. The 3D coordinates of these landmarks are then reconstructed using 2D positions from individual cameras by triangulation. System calibration is thus crucial to accurately collect data. Under optimal conditions, these systems have an excellent accuracy and precision, even in dynamic situations. These advantages make this technology currently the gold standard in biomechanical studies on SMR [8,9]. However, accuracy decreases with markers’ obstruction, which is inevitable during SMR training considering the numerous people needed to perform manoeuvres. Moreover, this technology is mainly restricted to laboratory use, reducing the variety of simulation scenarios that can be performed.

Inertial systems combine 3-axis accelerometers, measuring linear acceleration, and 3-axis gyroscopes, measuring angular velocity. Inertial measurement unit (IMU) also includes a magnetometer capturing the magnetic field. Based on these measurements, algorithms estimate the orientation of each module, expressed in a fixed global reference frame based on gravity and magnetic North. Even if accuracy and precision are usually sufficient for these types of applications, noise and error from these signals increase with integration and cause the drift of the position and orientation data. A ferromagnetic environment, and the type and speed of motion can also affect the accuracy [8,10]. Thus, it cannot be used for accurate measurement in every environment and for all simulations scenarios. Qualified staff are also needed to operate these systems and ensure the reliability of measurement. On the other hand, IMUs are small, portable, and do not require a clear line of sight, which makes them a good choice outside of the laboratory.

Electromagnetic systems are composed of one electromagnetic field transmitter and multiple sensors. The sensors consist of three orthogonal coils that produce electrical current from the electromagnetic field from which position and orientation can be inferred. There is therefore no line of sight problem with this technology. However, in similar conditions, their accuracy is not as good as the optoelectronic system and may be affected by ferromagnetic metals. The capture volume is also limited by the relatively small transmission range of the transmitter [8]. It is thus hard to use this type of system in complex simulation scenarios where the patient must be moved around.

**Frank: an instrumented simulation mannequin.**

To overcome the various challenges for SMR scenarios reported in the previous sub-section, our research team has tried to use cadavers and instrumented simulation patients to study relative motion of the head to the trunk during simulation scenarios. These approaches presented limitations such as the body’s flaccidity, ethics approbation, and technical difficulties. Since no mannequin in the market proposed a good bio-fidelity in terms of motion, our team has developed Frank, a mid-fidelity instrumented simulation mannequin designed for emergency medical services training [11]. Frank is a full humanoid silicon-based shape bio-realistic mannequin measuring 170 cm and weighing 75 kg, proportionally distributed. Its internal articulated skeleton respects the anthropometry of an average American male. Based on cadaveric and anthropometric studies, all limbs are articulated and have the average weight, inertia, and expected range of motion (see video on Frank’s articulations accessible within the Supplementary Materials) [12,13,14,15,16,17,18]. Frank thus reproduces the biomechanics of an unconscious man. He is also equipped with an airway for intubation and ventilation and his chest is compressible to perform cardiopulmonary resuscitation (CPR).

Frank also has a spine that is instrumented at the cervical and lumbar levels. Each segment is composed of four joints: two in flexion, one in lateral flexion, and one in rotation. This configuration allows us to mimic the motion of the spine in all anatomical planes. Frank’s spine is thus meant to reproduce the movement of the spine at a segment level. It shall be mentioned that even though the centres of rotation are not identical to those of a true human spine, the rigidity of the tissues surrounding it conceals the discrepancies between the structures while moving the head. The palpation feeling is also not realistic due to the structural difference between Frank’s spine and humans’ spines. The clinical representativeness in terms of the segments’ motion and inertia has been evaluated and confirmed informally by a small group of experts (orthopedic surgeon, anaesthetist, sport therapist trainer, and physiotherapist) and through feedback from many (n>150) pre-hospital care and healthcare workers (firefighters, paramedics, ski patrol, sports therapist, nurses, and trauma doctors) during extensive use of the mannequin in usability tests. Magnetic encoders at each joint measure angular position. This type of encoder includes a magnet installed on the shaft of the joint and a Hall effect sensor fixed on the shaft’s axis. The sensor detects the orientation of the magnetic field and infers the corresponding angular position with a claimed precision of 0.0219° [19].

Based on the previously introduced limitations regarding cadaver-based, simulated patients and currently available mannequin approaches, Frank appears as a potential improvement to objectively assess SMR manoeuvres during training as well as in research contexts. However, motion accuracy, referenced anatomically, shall be validated before Frank can be used in different contexts. Accordingly, the objectives of this study are (1) to assess the accuracy of the mannequin’s measurements in comparison with a gold standard, and (2) to validate that motion speed has no impact on accuracy.

## 2. Materials and Methods

The methodological section first describes the anatomical model used with the instrumented spine of the mannequin to allow the conversion of the data provided by the encoders into an anatomically referenced motion, hence facilitating clinical interpretation of the effectiveness of the SMR. The detailed experimental protocol used to collect data for cervical and lumbar motion validation is then presented. Details on data reduction and analysis leading to the comparison of motion measurements follow.

### 2.1. Anatomical Model Definition

#### 2.1.1. Cervical and Lumbar Assembly Model

To achieve clinical meaning, encoders’ data must be transformed into the relative orientation of the head to the trunk, herein referred to as the cervical spine, and the relative orientation of the trunk to the pelvis, also called the lumbar spine in the present context. This process is based on a robotic approach called forward kinematics, which is aimed at determining the position and orientation of the end-effector, knowing the values at each joint. For Frank’s cervical spine, the head represents the end-effector, and the trunk, the base. Forward kinematics can therefore be used for Frank’s spine since the values of each revolute joint are given by an encoder.

A forward kinematic model usually requires us to define six parameters for full position and the orientation definition of a link relative to the previous one: three in rotation and three in translation. However, applying conventions onto the coordinate system allows us to cut down the number of required parameters. Under the widely-used Denavit–Hartenberg convention in forward kinematics, the coordinate frames are linked to the joint between two links rather than the links themselves. It specifies the following:

1. The $z_{i}$ axis must be located along the axis of the joint *i*.
2. The $x_{i}$ axis must be normal to the $z_{i}$ and $z_{i-1}$ axis. If $z_{i}$ and $z_{i-1}$ are parallel, the direction of $x_{i}$ is chosen arbitrarily.
3. The $y_{i}$ must be defined with respect to the right-hand rule.

Applying these rules reduces the required number of parameters to four to fully define the coordinate system of each link:

- The joint length, $a_{i}$, defined as the distance between the $z_{i-1}$ and $z_{i}$ axis, along the $x_{i}$ axis.
- The joint twist, $\alpha_{i}$, defined as the angle between the $z_{i-1}$ and $z_{i}$ axis, along the $x_{i}$ axis.
- The joint offset, $d_{i}$, defined as the distance between the $x_{i-1}$ and $x_{i}$ axis, along the $z_{i-1}$ axis.
- The joint angle, $\theta_{i}$, defined as the angle between the $x_{i-1}$ and $x_{i}$ axis, along the $z_{i-1}$ axis.

**Figure 1:**
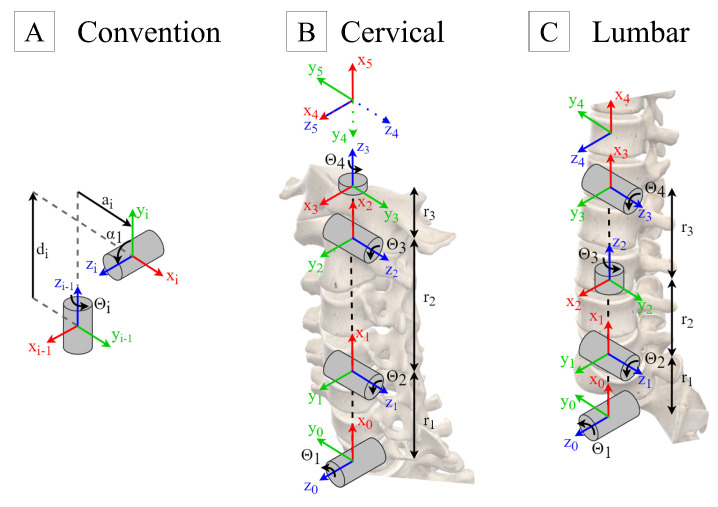
Frank’s spine models following Denavit–Hartenberg convention. (**A**) Denavit–Hartenberg’s rules. (**B**) Frank’s cervical Model. (**C**) Frank’s lumbar model.

Frank’s cervical and lumbar spine assembly was thus modelled following the Denavit–Hartenberg convention (panel A of Figure 1), resulting in the parameter values listed in Table 1. It is to be noted that the last coordinate systems, shown at the top of panels B and C of Figure 1, are created to ease the comparison of the end effector’s orientation to the base, since they have the same orientation in a neutral position.

From this analysis, transformation matrices between consecutive joints are derived. Equation (1) represents the transformation matrix from link i−1 to *i*, where *R* corresponds to the rotation and *r* to the translation. The global transformation matrix allows us to infer the relative motion in translation and rotation between the end-effector and the base, which can then be obtained by multiplying transformation matrices of all links, as presented in (2).

(1) $$\begin{array}{l} T_{i-1}^{i}=\left[\begin{array}{ccccc} & & & | & \\ & R_{i-1}^{i} & & | & r_{i-1}^{i}\\ & & & | & \\ 0 & 0 & 0 & | & 1 \end{array}\right]\\ =\left[\begin{array}{ccccc} \cos\theta_{i} & -\sin\theta_{i}\cos\alpha_{i} & \sin\theta_{i}\sin\alpha_{i} & | & a_{i}\cos\theta_{i}\\ \sin\theta_{i} & \cos\theta_{i}\cos\alpha_{i} & -\cos\alpha_{i}\sin\alpha_{i} & | & a_{i}\sin\theta_{i}\\ 0 & \sin\alpha_{i} & \cos\alpha_{i} & | & d_{i}\\ 0 & 0 & 0 & | & 1 \end{array}\right] \end{array}$$

(2) $$T_{1}^{n}=T_{1}^{2}T_{2}^{3}\ldots T_{n-1}^{n}$$

The global rotation matrix $R_{1}^{n}$ can then be transformed into three elemental and independent rotations, such as Euler angles, to describe the orientation of a body based on a known reference. In our case, the three axes of rotation are the anatomical axes, and the known reference is the neural position (Section 2.1.2). This representation has the advantage of being intuitive. However, when two rotational axes are lined up, one degree of freedom (DOF) is lost, causing a problem called gimbal lock. This phenomenon results in the loss of the ability to correctly describe the body orientation in three dimensions. To overcome this problem, a less intuitive representation can be used, the quaternions. A quaternion is composed of four components: three values to describe 3D vectors around which movement is performed, and a fourth value describing the angle of rotation of the body around this vector. Thus, the angle component of the quaternion can be used to describe the global range of motion of a body compared to its reference state, independently of the anatomical axes. To facilitate comprehension, both approaches are used in the current manuscript.

#### 2.1.2. Neutral Calibration

**Figure 2:**
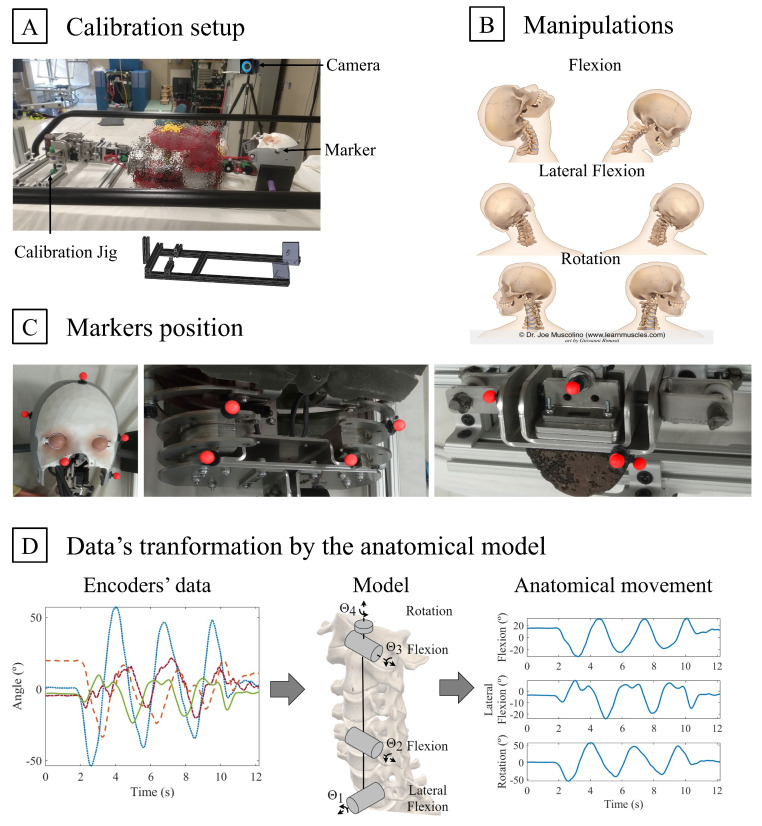
Data collection for Frank’s validation. (**A**) **Calibration setup** with Frank’s spine placed in neutral position in the calibration jig. (**B**) **Manipulations** of the segment in the three anatomical planes. (**C**) **Marker’s position** of all three rigid bodies: head (**left**), trunk (**centre**), and pelvis (**right**). The markers’ colour was changed to help visibility. Two markers on the pelvis are not visible on this picture. (**D**) **Data transformation by the anatomical model:** encoders’ data from combined trial are put in the anatomical model to obtain anatomical motion data. These data are then compared to data from optoelectronic system to optimize the alignment and the initial values of the model.

In order to represent the movement in anatomical planes, a neutral anatomical calibration is required. In biomechanical studies, the subject is typically asked to stand in an upright position looking forward. Due to the impossibility to maintain the mannequin in a perfectly upright position, a jig has been designed to position the mannequin in a neutral supine position (panel A of Figure 2).

### 2.2. Data Collection

The validation of Frank’s body’s orientation is achieved through concomitant comparison of the orientation data assessed with Frank to a gold standard motion capture system. The motion capture system used in this protocol is an optoelectronic system including 8 Optitrack (NaturalPoint Inc., Corvallis, OR, USA) cameras (6 $Prime^{X}13$ with 0.2 mm 3D accuracy and 2 $Prime^{X}13W$ with 0.3 mm 3D accuracy). Cameras were positioned to track passive markers in a 3 m × 2 m × 1.5 m capture volume with an accuracy of 0.688 mm, as assessed through Optitrack calibration protocol. Markers’ positions were tracked using Optitrack’s proprietary software, Motive 2.1.1. Specifically, markers were apposed on Frank’s head, trunk, and pelvis to create rigid bodies to be tracked in position and orientation (panel C of Figure 2). The head, trunk, and pelvis’ rigid bodies included 5, 4, and 6 markers, respectively. It is to be noted that a smaller number of markers were used for the trunk since this segment remained static during the manipulations. Markers were placed asymmetrically to ensure optimal tracking and reconstruction of the rigid body.

Optitrack’s ground plane was placed next to the jig to align the two measurement systems. Rigid bodies were created with Frank placed in the jig, in a neutral position, and encoders were set to zero. The mannequin was then removed from the jig for the rest of the protocol. In order to minimize the global bias between the two measurement tools, an alignment optimization was conducted between Optitrack and the encoder’s value (panel D of Figure 2). One trial per mannequin’s segment, moved in all three anatomical planes at the same time, was used to perform this optimization process.

Each trial was initiated with Frank resting in a neutral supine position. The protocol included movement along the major axis in each plane of motion (panel B of Figure 2) as well as combined motions, for a total of four conditions at both cervical and lumbar levels. Trials were composed of three cycles of full-range movement, finishing in a neutral position. Each condition was repeated ten times, five at fast speed (1 s/cycle) and five at slow speed (4 s/cycle). A grand total of 80 trials (40 per segment) were thus acquired with the same operator moving the segments. Throughout data acquisition, great care was taken to minimize obstructions of the markers to the cameras (e.g., specific hand and body placement chosen). A video illustrating the concept of the manipulations is accessible through the Supplementary Materials.

### 2.3. Data Reduction and Analysis

Data processing and analysis were performed in Matlab R2020b (Mathworks, Natick, MA, USA).

#### 2.3.1. Data Pre-Processing

Data pre-processing includes systems synchronization and outlier analysis. The mannequin and the optoelectronic system were first synchronized using cross correlation on the signal from the anatomical plane with a greater range of motion. To standardize this, one second in a neutral position was kept prior to the movement initiation. Outliers were detected from angular velocity: an instantaneous global motion velocity greater than 120°/s was flagged as an outlier and removed from the signal. If there was less than 50% of outliers in a 0.2 s time span, the points of this window were interpolated to reconstruct the signal. This approach ensures reliability of the gold standard which can be momentarily imperfect due to marker obstruction.

#### 2.3.2. Performance Markers

Accuracy is defined as the “closeness of agreement between a quantity value obtained by measurement and the true value of the measurand [20]”. In the current protocol, the movement assessed by the motion capture system is considered the true value. Accuracy is evaluated using the root-mean-square error (RMSE) between the motion assessed with Frank and the motion-capture gold standard system (3). Interpretation of the RMSE value in the context of use follows the guidelines proposed by McGinley et al., presented in Table 2 [21]. It is important to note that these guidelines refer to accuracy within a context of use, and therefore goes beyond the actual sensor accuracy. These values include errors on sensor measurement, imperfection in anatomical calibration, environmental perturbations, etc. Thus, resulting guidelines may appear high, but are, in fact, realistic and judged tolerable in an SMR context. In fact, 5° to 10° motion in the cervical spine is difficult, if not impossible, to detect with the naked eye. According to a study by Shrier et al., 44% of the logroll trials deemed perfect by a simulated patient corresponded to a cervical motion between 5° and 10°, whereas 16% were linked to a relative motion over 10° [4].

(3) $$RMSE=\sqrt{\frac{\sum_{i=1}^{n}{\left(x_{1,i}-x_{2,i}\right)}^{2}}{n}}$$

The optoelectronic system is recognized in the literature as a gold standard, though careful considerations must be made at different levels to ensure constant fidelity, as introduced in Section 2.3.1. Although best care was taken in this protocol to minimize the risks, the level of error involved can challenge the idea of absolute truth. As such, another method based on interchangeability, the Bland–Altman method, was used to complement accuracy assessment. Unlike RMSE, the Bland–Altman method does not consider one system as perfect: it quantifies the agreement between two measurements tools, usually an accepted technology and a new one. The quantification of the agreement is performed by computing a bias and a limit of agreement. This method requires the differences to be normally distributed which can be verified using a Shapiro–Wilk test. To ensure the equal representativeness of fast and slow trials, 10 points were randomly selected, per trial, to be used in the Bland–Altman analysis. Guidelines used for RMSE interpretation are also used to interpret the confidence interval obtained with the Bland–Altman analysis.

The minimal detectable change (MDC) is an estimate of the smallest amount of change that can be detected without doubt that this change is not due to measurement error. The MDC is calculated based on a 95% confidence interval and a standard error of measurement (SEM). SEM estimates the variation around a score if the test is repeated multiple times; it is based on a reliability coefficient. In this protocol, the reliability coefficient was calculated based on a parallel forms reliability method [22]. Essentially, a correlation coefficient between the scores of two different but equivalent tests is computed. Herein, the score refers to the motion value (global or in a specific anatomical plane), whereas the tests refer to the systems (i.e., the mannequin and the motion capture system). Again, this correlation coefficient was computed from 10 random samples for each trial. The guidelines exposed in Table 2 are also used to interpret the MDC.

To verify that the motion velocity does not have an impact on accuracy, a non-inferiority analysis is conducted on the RMSE values, using a one-sided *t*-test. Type I error level α is fixed to 2.5% and the chosen threshold corresponds to the identified MDC value. We cannot consider that the speed has an influence on the accuracy if the RMSE difference between the fast and the slow motion is below this value.

## 3. Results

A total of 80 trials were conducted, 40 on each level of Frank’s spine. However, five trials from lumbar spine validation (one in flexion, three in rotation and one combined movement) were discarded due to a prolonged loss of the pelvis rigid body within the reference motion capture system. Overall, the results are reported on 40 trials for the cervical spine and 35 for the lumbar spine.

### 3.1. Mannequin’s Accuracy

**Figure 3:**
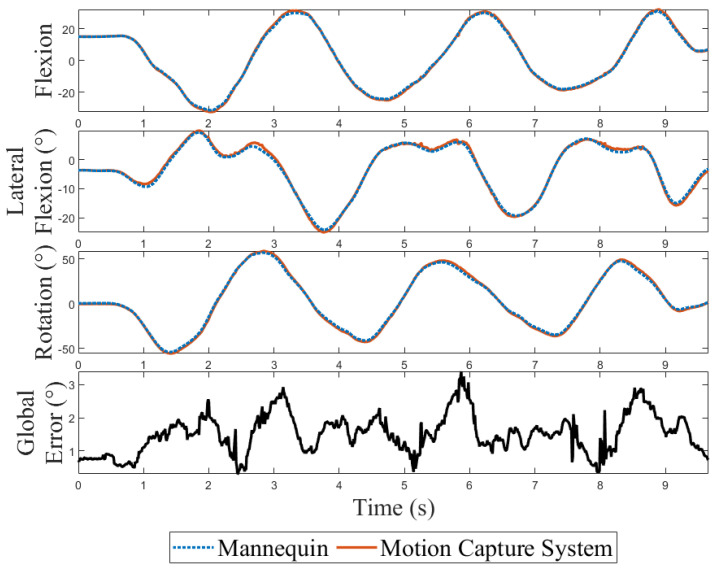
Signal comparison of the mannequin and the motion capture system in all three anatomical planes. The global error between the two measurement tools is also displayed.

Figure 3 illustrates motion recorded by both systems during a typical combined motion trial. An analysis of the error revealed a global mean RMSE of 1.9° for the cervical spine and 2.3° for the lumbar spine, with a variation between 0.7° and 1.5° among the anatomical planes of motion (Table 3).

**Figure 4:**
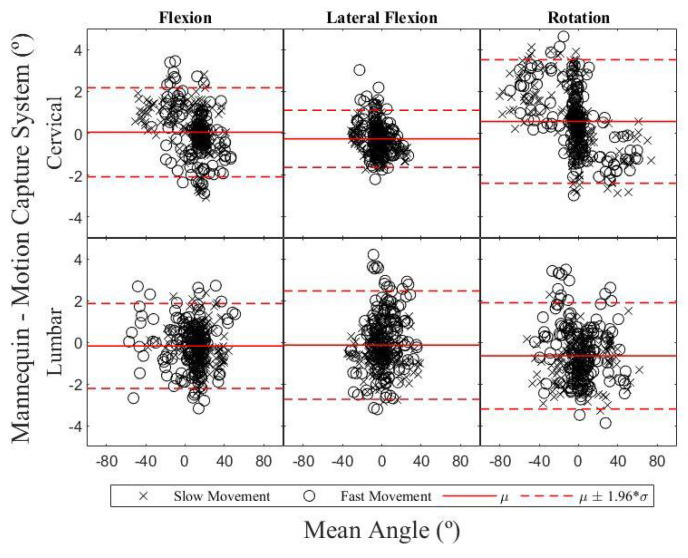
Bland–Altman plot of the two segments in the three anatomical planes of motion, in which the Y axis represents the differences between paired values of the two measurement tools and the X axis represents the mean of both values.

An analysis of interchangeability between the two measurement systems is illustrated using the Bland–Altman method in Figure 4, while Table 4 lists the resulting biases between the two measurement systems, per anatomical planes of motion. The differences were approximately normally distributed (W > 0.96) allowing us to compute the 95% confidence intervals for the limits of agreement, also reported in Table 4. For the cervical spine, the bias varied between −0.3° and 0.6° with the absolute limits of agreement remaining below 3.5°. For the lumbar spine, the bias varied between −0.7° and −0.1°, whereas the absolute limits of agreement also remained below 3.5°.

The reliability coefficient (r) between the measurement systems remained above 0.998 for the cervical spine and 0.996 for the lumbar spine in all planes of motion (Table 5). Thus, the MDC for the cervical spine varied between 1.3° and 2.4° for the cervical spine and between 2.0° and 2.6° for the lumbar spine.

### 3.2. Influence of Motion Speed on Accuracy

The non-inferiority test between the RMSE for fast and slow motion trials using a 2.05° threshold for the cervical spine confirmed the rejection of the null hypothesis (p<0.001). For the lumbar spine, the same conclusion is obtained (p<0.001) using a threshold of 2.45°.

## 4. Discussion

The current study was aimed at defining the movement accuracy of a simulation mannequin at cervical and lumbar levels. At the cervical level, RMSE values remained below 2°, which correspond to a good accuracy based on the guidelines suggested by McGinley et al. [21]. At the lumbar level, accuracy was good for all planes of motion, but acceptable at the global level. Keeping the same guidelines to evaluate the 95% confidence intervals computed with the Bland–Altman method, all values were acceptable, except for the lateral bending at the cervical level which was good. The obtained MDC values are considered acceptable to good, again in regards to McGinley et al.’s guidelines.

In the literature, Conrad et al. analyzed different techniques of transfer from a prone to a supine position on cadavers with an unstable spine using an electromagnetic systems [5,6]. These studies considered a small sample of highly qualified experts in constrained conditions. The authors report a significant difference of 1° in lateral flexion for the cervical spine and 3° in flexion at the lumbar level. According to the MDC assessed for Frank, the difference could be captured using this new mannequin. Yet, the limited number of highly qualified experts reduces the variation and therefore constitutes some of the most difficult conditions with regards to difference detection. One can also question the external validity of the results within a representative population of first responders. Frank was designed to be used outside the laboratories and be manipulated by a wide range of health-care professionals. Therefore, Frank’s accuracy is considered sufficient to capture the desired variation in data motion to be collected, while allowing us to get out of constrained laboratory conditions. In fact, no change in accuracy is foreseen in realistic scenarios due to the nature of the sensors used.

The current study was also aimed at validating that motion velocity has no impact on accuracy. With *p*-values below 0.001 for both cervical and lumbar tests, conducted using MDC as thresholds, the null hypothesis can be rejected for both segments. We can therefore conclude that speed has no effect on the measurement accuracy of the mannequin. Frank can thus be used in all sorts of simulation scenarios in the field unlike other measurement tools, like inertial unit, which have shown reduced accuracy while measuring fast movements [10].

A potential limitation of the study is that Frank’s cervical spine motion accuracy seemed better than its lumbar spine, while it was expected to be similar. Two hypotheses are raised by the authors to explain that difference. First, the mechanical structure of the lumbar is looser than the cervical spine due to the mechanical design. Therefore, a portion of the movement might have been lost in the mechanism and thus not be read by the encoders. Improvements in the mechanical design will be made in the next version of the mannequin to address this issue. The second hypothesis relates to the potential impact of marker obstruction. Noise-like oscillations in the motion capture system signal were observed near the maximum amplitude of the movement (Figure 3). Since it is the place where obstructions are most likely to happen because rigid bodies were created in a neutral position, oscillations are thought to be caused by this phenomenon. Furthermore, more obstructions were observed during lumbar movements due to the structural shape of the pelvis. Because marker obstructions increase the error of the motion capture system measures, this could have artificially increased the error between the two measurement systems, thus causing this difference in the results.

It is also to be noted that alignment and calibration were optimized to minimize the bias between both measurement systems while performing a full range movement of a segment in all anatomical planes. However, if marker obstruction had caused an error in the signal acquired with the motion capture system, it may have caused an error in the optimization process. As the mannequin’s desirable use is around its neutral position, optimizing the alignment and calibration parameters around the neutral position might be preferable.

Based on these validation results, we can conclude that Frank is a good measurement tool to assess the head to trunk motion of an unconscious patient. Its accuracy, comparable to other motion capture tools, is sufficient to measure and compare different techniques. Nevertheless, it should be emphasized that due to the distinctiveness of Frank’s spine structures compared to those of a human being, the palpation feeling will not accurately replicate real-life sensations. Despite this, the present study sought validation from a diverse group of pre-hospital and healthcare professionals in an informal manner, confirming Frank’s bio-realistic sensation. Future studies will incorporate a more structured approach to assess the mannequin’s motion bio-representativeness. This evaluation will be conducted qualitatively, seeking experts’ impressions within specific contexts of use to ensure a comprehensive and formal understanding of Frank’s performance. This study will also be useful to test Frank’s robustness in a wide variety of simulation scenarios. Therefore, Frank could be used where a simulated patient could not be for safety or ethical reasons. Frank’s ability to assess motion could also be useful for health care professionals’ training. Indeed, data from spine motion can help us to identify the best techniques but also pinpoint where a potential problem may have occurred. Along that line, a next logical step with Frank is to define feedback metrics rather than just providing the range of motion of a spine segment during a manoeuvre, to help understand the cause of the undesired movement. Having metrics, such as delay at the start of a manoeuvre, is foreseen to allow the trainees to better understand the required manipulations and synchronization to perform a good mobilization and transfer process to reduce the motion of the patient’s spine.

## 5. Conclusions

The results presented in this paper open the possibility to use Frank, a mid-fidelity instrumented mannequin, in diverse simulation scenarios for SMR training and research studies. Frank’s demonstrated accuracy is sufficient to reach the same conclusion as other SMR studies performed in highly constrained conditions. However, Frank is designed to enable its use outside the laboratories, in less constrained environments, and under more representative scenarios. A qualitative clinical validation in the context of use will be performed next to demonstrate that Frank is a good alternative to measure spine motion in a wide range of conditions, thanks to its versatility.

## Figures and Tables

**Table 1:** Denavit–Hartenberg Model Parameters.

	Cervical				Lumbar			
**Joint**	a	α	d	θ	a	α	d	θ
**1**	$r_{1}$	π/2	0	$\theta_{1}$	$r_{1}$	π/2	0	$\theta_{1}$
**2**	$r_{2}$	0	0	$\theta_{2}$	0	π/2	0	$\theta_{2}+\pi /2$
**3**	0	π/2	0	$\theta_{3}+\pi /2$	0	−π/2	$r_{2}+r_{3}$	$\theta_{3}$
**4**	0	−π/2	$r_{3}$	$\theta_{4}$	0	−π/2	0	$\theta_{4}-\pi /2$
**5**	0	−π/2	0	−π/2				

**Table 2:** Accuracy interpretation.

RMSE Values	Accuracy Interpretation
RMSE ≤ 2°	Good
2° < RMSE ≤ 5°	Acceptable
5° < RMSE ≤ 10°	Tolerable
RMSE > 10°	Unbearable

**Table 3:** Root-mean-square error.

	Cervical		Lumbar	
	**RMSE (°)**	**SD (°)**	**RMSE (°)**	**SD (°)**
**Global**	1.9	0.5	2.3	0.5
**Flexion**	1.1	0.3	1.1	0.2
**Lateral Flexion**	0.7	0.2	1.3	0.5
**Rotation**	1.5	0.5	1.5	0.4

**Table 4:** Bland–Altman results.

	Cervical		Lumbar	
	**Bias (°)**	**CI_95_ (°)**	**Bias (°)**	**CI_95_ (°)**
**Flexion**	0.0	−2.2–2.2	−0.2	−2.3–1.9
**Lateral Flexion**	−0.3	−1.7–1.2	−0.1	−2.8–2.6
**Rotation**	0.6	−2.4–3.5	−0.7	−3.3–2.0

**Table 5:** Minimal detectable change.

	Cervical				Lumbar			
	**r**	**SD (°)**	**SE_m_ (°)**	**MDC (°)**	**r**	**SD (°)**	**SE_m_ (°)**	**MDC (°)**
**Global**	0.999	19.7	0.7	2.1	0.997	17.5	0.9	2.5
**Flexion**	0.998	17.3	0.7	1.9	0.998	17.6	0.7	2.0
**Lateral** **Flexion**	0.998	10.0	0.5	1.3	0.996	14.1	0.9	2.6
**Rotation**	0.999	25.2	0.8	2.4	0.997	19.0	0.9	2.5

## Data Availability

Data is available upon request to the authors.

## Supplementary Materials

The following supporting information can be downloaded at: https://www.mdpi.com/article/10.3390/s24041055/s1, Video S1: Articulations 1; Video S2: Manip 1.

## Abbreviations

The following abbreviations are used in this manuscript:

SMR	Spinal motion restriction
SCI	Spinal cord injury
IMU	Inertial measurement unit
CPR	Cardiopulmonaty resuscitation
RMSE	Root-mean-square error
MDC	Minimal detectable change
